# A model of the evolution of equitable offers in n-person dictator games with interbirth intervals

**DOI:** 10.1038/s41598-021-94811-3

**Published:** 2021-07-30

**Authors:** Jeffrey C. Schank

**Affiliations:** Department of Psychology, University of California, Davis, Davis, CA USA

**Keywords:** Evolution, Social evolution, Psychology, Human behaviour

## Abstract

People are often generous even when it is not in their apparent self-interest to do so as demonstrated by numerous experiments using the dictator game (DG). More recent research using DGs has varied the number of dictators and recipients and used these games to investigate the bystander effect and congestible altruism. These studies have found that people are less generous when there are others who could be generous (the bystander effect) and also less generous when there are multiple recipients (congestible altruism) though the sum of their generosity to all recipients increases. A working hypothesis is proposed that the context-sensitive generosity observed in *n*-person DGs can be explained as equitable behavior. From an evolutionary perspective, explaining the evolution of equitable behavior is challenging at best. To provide an evolutionary explanation, a second working hypothesis is proposed: equitable offers evolve because they reduce resource deficits produced by variability in the accumulation of resources and thereby minimize the length of interbirth intervals (IBIs) and increase fitness. Based on this working hypothesis, an evolutionary model was developed for *n*-person DGs to investigate the evolution of equitable offers as a resource allocation problem when reproduction is constrained by IBIs. Simulations demonstrated that equitable offers could evolve in group-structured populations when there is a cost (i.e., longer IBIs) to running resource deficits. Mean evolved offers also varied as a function of the number of dictators and recipients in patterns consistent with the bystander effect and congestible altruism. Equitable offers evolved because they reduced resource variability among group members and thereby reduced resource deficits, which insured higher average rates of reproduction for more equitable groups of agents. Implications of these results are discussed.

## Introduction

People are often generous even when it is not in their self-interest as demonstrated by numerous experiments using the dictator game (DG). In the classical form of the DG there are two players, a dictator and a recipient. The dictator decides how much of a resource endowment to offer a recipient and the recipient must take whatever the dictator offers. Typically, players are randomly paired and anonymous when the offers are made. Not surprisingly, the self-interested solution is for the dictator to offer nothing, but numerous experimental studies have found that, on average, dictators often make generous offers^1,2^. A 2011 meta-analysis of the DG found that dictators offer on average over 28% of an endowment^2^ and a large study conducted across 15 societies found that dictators offered, on average, 37% of an endowment with a range from 26 to 47% across societies^3^.

A DG can consist of more than one dictator and more than one recipient. Such *n*-person DGs have been used to investigate different types of generous or altruistic behaviors. In *n*-person DGs, the generous behavior displayed in classical DG experiments varies as the number of players and their roles varies. For example, DGs with multiple recipients have been used to investigate congestible altruism, the phenomenon that generosity decreases as the number of recipients increases^4–8^. More specifically, the size of offers made by dictators is congestible and so offers decrease as the number of recipients increases, but the *total offer* (i.e., the sum of the offers made to recipients by a dictator) increases as the number of recipients increases.

In contrast, to multi-recipient DGs, there are multi-dictator games, which have been used to investigate the bystander effect. The bystander effect is the phenomenon that a person is increasingly less likely to help another person in the presence of bystanders^9^. Early research on the bystander effect in the context of altruistic giving found that how much a person donated to victims of a catastrophe decreased as group size increased^10^. In subsequent experimental studies using the DG with multiple dictators, the size of offers made by dictators decreased as the number of dictators increased^8,11^.

Years of research with *n*-person DGs has yielded two general results: (i) people are more generous than predicted by self-interest alone and (ii) their generosity varies with the number of dictators and recipients. For congestible altruism, while dictator offers to individuals decrease as the number of recipients increase, the sum of offers dictators make increases as the number of recipients increases. For the bystander effect, offer size decreases as the number of dictators increases. The first working hypothesis of this paper is that the context-dependent generosity observed in *n*-person DGs can be explained by people behaving equitably and a simple example illustrates the plausibility of this hypothesis.

Consider how offers change as the number and roles of participants in an *n*-person DG change if the goal is to achieve an equitable distribution of resources. If *k* is the number of dictators in an *n*-person DG, then *kR* is the total resources to be distributed among the *n* players (i.e., each dictator receives a resource endowment *R* to start a game). An equal distribution of resources among players requires *kR*/*n* resources for each of the *n* players. This can be achieved if dictators offer recipients *pR*, where *p* = 1 − *k*/*n* and *p* is the portion of the resource endowment offered by a dictator to recipients. Assuming offers made by dictators are evenly split among the (*n* − *k*) recipients, then values of *p* that achieve an equal distribution of resources among *n* players are for one dictator and one recipient, *p* = 1/2, for one dictator and two recipients, *p* = 2/3, and for two dictators and one recipient, *p* = 1/3. Notice that as the number of recipients increases, *p* increases but as the number of dictators increases, *p* decreases. This example illustrates that if people do tend to behave equitably, then the size of the offers they make should change as the number of dictators and recipients changes. On this interpretation, the generous offers made by dictators in *n*-person DGs are *equitable offers*.

The most difficult challenge remains: why do people often behave equitably in DGs when it is not in their apparent self-interest? From an evolutionary perspective, the kind of equitable behavior observed in *n-*person DGs appears to be implausible at best. Consider a population where individuals reciprocate equitable offers. In the case of one dictator and one recipient, the benefit of an offer to a recipient is *pR*, while the cost to a dictator is also *pR*. Even in the best-case scenario where all individuals always play the same equitable offer strategy, the benefits do not exceed the costs. There is no net benefit to reciprocal exchanges of resources even if there is no defection. A similar point can be made for kin selection or for other standard mechanisms for the evolution of cooperation, which require that the benefits must exceed the costs among cooperators^12^. Thus, it appears theoretically impossible for equitable offers to evolve.

To show that it is possible for equitable offers to evolve and do so in a pattern consistent with empirical results from *n*-person DGs requires a second working hypothesis: equitable offers evolve because they reduce resource deficits produced by variability in the accumulation of resources and thereby minimize the length of interbirth intervals (IBIs—the time required between the birth of offspring due gestation, lactation, and parental effort^13^) and increase fitness. In the model presented below, two basic assumptions were made: resources are accumulated by playing DGs and the production of offspring is constrained by IBIs. Repeatedly playing DGs introduces variability into the accumulation of resources, which can lengthen IBIs via resource deficits. Equitable offer strategies are shown to provide a mechanism for optimizing reproduction by reducing resource deficits, which minimize IBIs and thereby increase fitness.

**Figure 1:**
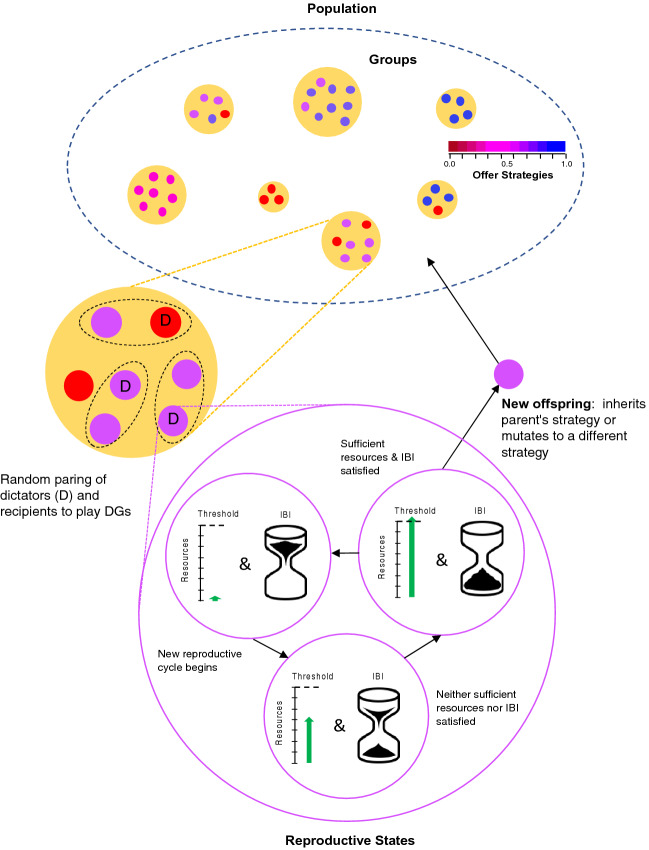
An illustration of the IBI model of the evolution of equitable offers strategies in *n*-person DGs. At the top is a population consisting of groups with individual agents color coded by strategy. Some agents in each group are randomly assigned a resource endowment and then agents are randomly assigned to play DGs within a group (dashed ovals in the magnified group indicate DGs). Agents accumulate reproductive resources (green) from playing DGs and when an agent has accumulated sufficient resources (reaches or passes the resource threshold) and the IBI is complete (“hourglass” is finished), an offspring agent is born, which either inherits the parent’s offer strategy or mutates to a different offer strategy. Resources required to reproduce are subtracted and the IBI is reset to its initial state (“hourglass” is flipped over).

The optimizing effect of reducing variation can only occur if equitable agents can non-randomly assort with each other. One mechanism for achieving assortment is population structure^12^. For a population structured by groups, some agents are randomly assigned a resource and are dictators while the remaining agents are recipients (Fig. 1). On each time step, agents are randomly assigned to play DGs (Fig. 1). Agents accumulate resources over time by playing DGs and reproduce when they have accumulated sufficient resources and sufficient time has passed as determined by the length of their IBIs (Fig. 1). In the model presented here, I show equitable offer strategies can optimize the conversion of reproductive resources into offspring in populations structured by groups when there is a cost to running resource deficits caused by resource variation.

## Model and results

In the sections below, I begin by describing the behavior of agents that play DGs and then turn to the problem of how agents optimally allocate the resources they accumulate to produce offspring under the constraint of IBIs. I then analyze the variation in accumulated resources that emerges from repeatedly playing DGs. This analysis reveals that patterns of variation in accumulated resources change as the number of dictators and recipients changes in *n*-person DGs. These patterns of variation reveal that different offer strategies achieve resource equitability as a function of the number of dictators and recipients in a DG. I next introduce a resource deficit model in which IBIs lengthen as a consequence of running resource deficits caused by resource variation. Finally, I show that equitable offer strategies can evolve—in populations structured by groups (see Fig. 1)—by minimizing resource deficits via increasing resource equitability and that strategies evolve in patterns consistent with results from classical 2-person DGs as well as for congestible altruism (more recipients than dictators) and the bystander effect (more dictators than recipients) experiments.

### DGs and agent resource accumulation

An *n*-person DG (DG_*k*,*n*_) consists of *k* dictators and *n* − *k* recipients (see “Discussion” for interpretations of agents). On each time step, a proportion *k*/*n* of the agents are randomly assigned as dictators and the remaining 1 − *k*/*n* agents are recipients. Each dictator receives a resource endowment *R* (i.e., resources devoted solely to producing offspring) and recipients receive nothing. Dictators offer a portion *p* (0 ≤ *p* ≤ 1) of *R* to recipients, where *pR* is the total offer made to all recipients in a game and the recipients evenly divide the offer, *pR*/(*n* − *k*). Agents accumulate resources *x* by participating in a DG_*k*,*n*_ on each time step as defined by Eq. (1).

1 $${x}_{t+1}=\left\{\begin{array}{l}{x}_{t}+R-pR,\quad dictator \\ {x}_{t}+\frac{\sum_{i=1}^{k}{p}_{i}R}{n-k},\quad recipient,\end{array}\right.$$

where *x*_*t*_ = 0 at *t* = 0 or at birth and, as a recipient, an agent sums the offers from *k* dictators each with their own offer strategies *p*_*i*_. Because the total resources available on each time step is *kR* and the total number of agents is *n*, the expected return for an agent participating in a game is *kR*/*n*.

### Optimal allocation of resources for reproduction

So far, I have defined how agents repeatedly play *n-*person DGs and accumulate resources over time to reproduce. The next step is to model how agents convert resources they acquire from repeatedly playing *n-*person DGs into offspring under the constraint of IBIs. Because this is an optimization problem, the appropriate starting point is optimal reproductive resource allocation theory^14^, which concerns how resources are optimally allocated among three demands: growth, body maintenance, and reproduction. As with most evolutionary game-theoretical approaches to cooperation, I assumed that all resources accumulated by repeatedly playing games are devoted to reproduction (though resources for survival are also important^15^, but not investigated here) and that the optimization problem for agents is to convert these resources into the maximum number of offspring given the expected resources obtained from repeatedly playing DGs.

On the assumption that agents obtain resource by playing DGs, the expected total resources accumulated over a lifetime is the expected return from playing a DG_*k*,*n*_ (i.e., *kR*/*n*) at each time-step times the total number of time-steps in a lifetime. If the lifetime of an agent is *ω* (measured in time steps), then the expected total resources accumulated over a lifetime is *ωkR*/*n*. If *I*_*o*_ units of resources are required to produce an offspring then the optimal number of offspring, *m*, that an agent can be expected to produce in a lifetime is its accumulated lifetime resources (*ωkR*/*n*) divided by the units (*I*_*o*_) required to produce an offspring, which is given by Eq. (2)

2 $$m={}\omega\frac{kR}{n{I}_{o}}.$$

Reproductive biology in mammals and other vertebrates places a time constraint (i.e., IBIs) on the optimal allocation of reproductive resources. IBIs constrain the rate of reproduction and are the result of processes such as gestation, lactation, and parental care^13^. Gestation places a minimal bound on IBIs and in humans gestation is approximately nine months^16^. Periods of deprivation can affect hormonal and physiological mechanisms that reduce the likelihood of pregnancy and thereby lengthen IBIs^17,18^. Gestation can be shorter than the typical nine months with resource scarcity but it results in lower offspring viability^18^. IBIs are, at a minimum, the length of gestation and typically much longer in natural fertility populations due to extended parental care, in particular, lactation^13,16^. Modulation of IBIs by resource scarcity and variability has arguably been critical for the evolution of human social behavior^19–21^.

The optimal number of offspring, *m*, can be achieved under the constraint of IBIs if the duration of the IBI*τ*_0_ coincides with the accumulation of *I*_*o*_ resources. On the assumption that selection optimizes the length of *τ*_0_ to match the expected accumulation resources required to produce an offspring (*I*_*o*_), *I*_*o*_ can be defined in terms of *τ*_0_ as in Eq. (3)

3 $${I}_{o}={}\tau_{0}\frac{kR}{n}.$$

To the extent that an agent fails to evenly distribute its available resources over its IBIs, it can suffer decreased fitness via longer IBIs. Note that as defined, *I*_*o*_ is in units (*kR*/*n*) of playing *τ*_0_ games. To avoid possible discrete effects of assuming the expected return of each game is a multiple of *I*_*o*_, normal random variation was introduced into the resource endowment *R* for each dictator in each game (see “Methods”, Simple Agent Model).

### Variation in accumulated resources

As defined above, while an agent accumulates reproductive resources at an expected rate of *kR*/*n* per time step, there is also resource variation generated over time based on the offer strategy it uses and the strategies used by other agents. Playing DGs introduces variation into the accumulation of resources and does so as a function of the offer strategies used by agents. For populations in which agents use different offer strategies, the resource variation generated over time is not analytically tractable. However, if all agents in a population play the same offer strategy (i.e., the population is homogenous with respect to offer strategy), then the variation in accumulated resources overtime can be analyzed as a function of offer strategies.

On each time step, I assumed that each agent is a dictator or recipient and that these roles are randomly determined as defined above. Because the roles of agents are randomly determined on each time step, the variance in resources on each time step is independent of previous time steps, which allows the calculation of the expected variation for a DG_*k*,*n*_ for each time step and for the accumulated variation over multiple time steps (see “Methods”, Resource Variation). A relative measure of resource variation under these assumptions is given by the coefficient of variation in Eq. (4).

4 $${CV}_{p,{\tau }_{0}}=\frac{n\sqrt{{\tau }_{0}\frac{k{R}^{2}{\left(k+np-n\right)}^{2}}{{n}^{2}(n-k)}}}{{\tau }_{0}kR}.$$

Equation (4) demonstrates that accumulated resource variation is minimized when agents use offer strategies that increase resource equitability among players. To illustrate this result, coefficients of variation were calculated for offer strategies in five different DG_*k*,*n*_ (i.e., DG_3,4_, DG_2,3_, DG_1,2_, DG_1,3_, and DG_1,4_), selected to represent cases from more dictators than recipients (i.e., bystander effect) to more recipients than dictators (i.e., congestible altruism).

**Figure 2:**
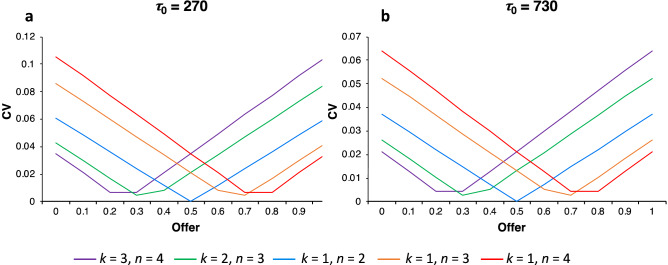
Accumulated resource variation as measured by coefficients of variation for the five DG_*k*,*n*_. For all but DG_1,2_, minimal coefficients of variation are not achieved because the minimum is achieved only when *p* = 1 − *k*/*n*, which does not always correspond to values in the range *p* = 0, …, 1.0 in increments of 0.1. Note that while accumulated resource variation for *τ*_0_ = 270 (**a**) and *τ*_0_ = 730 (**b**) have the same form, the range of the relative variation for *τ*_0_ = 270 is higher than for *τ*_0_ = 730.

Two sets of coefficients of variation were calculated for IBIs of *τ*_0_ = 270 (corresponding to an approximate 9-month gestation period, Fig. 2a) and for *τ*_0_ = 730 (corresponding to two-years of gestation, lactation, and parental effort, Fig. 2b), which assumes daily games (see “Discussion” for a discussion of the time units problem). More equitable offer strategies have lower coefficients of variation than less equitable offer strategies (Fig. 2a,b). However, the range of relative variation in resource accumulation decreases as *τ*_0_ increases from 270 to 730 (cf. Fig. 2a,b), which indicates that although variance in accumulated resources increases as *τ*_0_ increases, relative variation decreases.

Variability in the accumulation of resources has real-world consequences for reproduction. For example, in times of resource restriction, menstrual cycle variability among women increases and fecundity decreases, which contributes to variability in IBIs^17,18^. Evidence suggests that fetal loss, especially early in pregnancy, may be sensitive to resource availability as well as fetal growth and the resumption of fecundity postpartum^18^. Fecundity around the time of conception is especially sensitive to short-term changes in availability of resources^18^. Resource shortages can reduce resources available to nursing offspring and increase mortality. Thus, IBIs will vary and resource deficits can lengthen IBIs^13^.

### The cost of running resource deficits

A simple approach to modeling the effect of resource deficits is to introduce a time cost*, c* (0 ≤ *c* ≤ 1), for running resource deficits below a deficit-level cutoff *γ* (0 < *γ* < 1) in the expected accumulation of resources. For example, the duration of lactation is affected by the nutritional status of a woman, which in turn affects the resumption of menses and lengthen IBIs (Ellison, 1994). If *T* is the time since an agent last gave birth (or was born), then the expected accumulation of resources as time passes during an IBI is

5 $$\widehat{x}=T\frac{kR}{n},$$

where $\widehat{x}$ is the expected accumulation of resources and *T* = 1 after giving birth (or its own birth), otherwise *T* = *T* + 1. If the actual accumulation of resources (*x*_*t*_) falls sufficiently below the expected accumulation of resources (i.e., *x*_*t*_ < $\gamma \widehat{x}$), then a resource deficit occurs.

The optimal IBI that an agent can have in this model is *τ*_0_, but since resource deficits lengthen IBIs, a variable IBI, *τ*, is introduced such that *τ* = *τ*_0_ when an agent is born or after an agent gives birth, but lengthens with resource deficits that fall below $\gamma \widehat{x}$. If an agent runs a resource deficit, a portion of *τ*_0_ (*cτ*_0_) is added to *τ* as defined by Eq. (6), which assumes Eqs. (1) and (5) above

6 $$\tau =\left\{\begin{array}{ll}\tau +c{}\tau_{0}&\quad {x}_{t}<\gamma \widehat{x} \\ {\tau }_{0} &\quad \text{agent is born or gives }birth \\ \tau &\quad \mathrm{otherwise},\end{array}\right.$$

where *x*_*t*_ is an agent’s accumulated resources (see Eq. 1) at *t*. Thus, if at any given time step an agent runs a resource deficit, *τ* is increased according to Eq. (6).

The effect of running resource deficits are longer IBIs, which are caused, for example, by prolonged lactation or delays in the resumption of menses^13^. More sophisticated models could explicitly incorporate different kinds of time costs during different phases of reproduction, but the proposed simple model aims only to determine whether reducing resource deficits could favor the evolution of equitable offer strategies. This model does allow the accumulation of excess resources early during an IBI, but it does not allow resource deficits without lengthening an IBI. A lucky non-generous agent that accumulated abundant resources early in life could carry these excess resources throughout life and thereby produce an optimal number of offspring. However, a group of equitable agents could also insure running fewer resource deficits by more equitably distributing resources over time among group members and thereby minimize their IBIs and increasing their reproductive rate.

### Birth

Births occur when both sufficient resources have been accumulated and at least *τ* time steps have occurred since an agent was born or last gave birth as defined by Eq. (7)

7 $$birth=\left\{\begin{array}{ll}{\mathrm{true}} ,\quad {x}_{t}\ge {I}_{o}{{ \& }} T\ge \tau \\ {\mathrm{false}},\quad {\mathrm{otherwise}}.\end{array}\right.$$

The agent’s reproductive resources are then decremented by ${I}_{o}$ (i.e., the resources required to produce an offspring) as defined by Eq. (8)*.*

8 $${x}_{i,t}=\left\{\begin{array}{ll}{x}_{t}-{I}_{o},&\quad birth \\ {x}_{t}, &\quad \mathrm{otherwise}.\end{array}\right.$$

Finally, assuming a population of maximum size *N* (10,000 agents in these simulations). Reproduction was constrained analogous to a Moran process^22^, such that a newly born offspring enters the population only if the current population size is less than *N* otherwise the offspring agent “dies”.

Equation (8) allows for the complete carryover of any excess reproductive resources from the previous reproductive period. Allowing complete carryover could favor the non-generous strategy on occasions when more resources than expected are accumulated and carried over to the next reproductive period. Although more extensively studied in birds than mammals, resource carryover effects from one reproductive period or season to the next have been found to affect fitness^23,24^. Little is known about the quantitative effects of resource carryover on fitness in animals (O’Connor et al., 2014). In elk, the fecundity of cows and survival of calves are very sensitive to 10–20% carryover of the digestible energy content of food^24^. For human, if resource carryover was in a 10–20% range it could favor the evolution of more equitable offers because non-generous agents would be constrained in the quantity of resources that they could carryover. Resource carryover is not modeled here because of the lack of information, but constraining carryover would be biologically more realistic and likely favor more equitable strategies.

### Relative fitness of offer strategies in homogenous populations

If it is possible for equitable offer strategies to evolve, they must do better against themselves than less equitable strategies do against themselves because equitable offer strategies always do worse against less equitable strategies. Thus, simulations were run to assess the effects of resource variation on the relative fitness of different offer strategies played against themselves in homogenous populations (see “Methods”, Simple Agent Model).

First, I considered the situation of no cost (*c* = 0) to running a resource deficit. We see that variation in the accumulation of resources does play a role in the relative fitness differences among offer strategies (Supplementary Fig. S3). However, as IBIs increase in length, the effect of resource variation on relative fitness rapidly diminishes. That is, the range of relative fitness values decreased over four-fold from *τ*_0_ = 10 to *τ*_0_ = 270 (Supplementary Fig. S3a,b) as did the range of relative IBIs of these strategies (cf. Supplementary Fig. S3c,d). This is not surprising since the magnitude of relative variation decreases with increasing IBIs (see Fig. 2).

**Figure 3:**
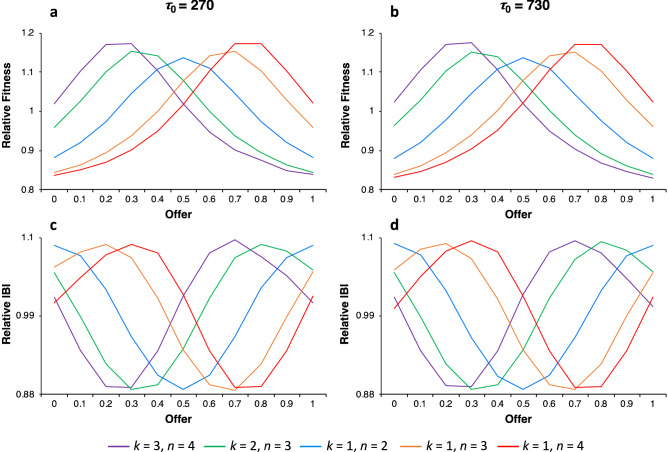
The effects of accumulated resource variation on the relative fitness and the relative IBIs of offer strategies for the five DG_*k*,*n*_ for *c* = 0.01 and *γ* = 0.9. Plots of the relative fitness values for offer strategies are nearly the same for *τ* = 270 (**a**) and *τ* = 730 (**b**). A similar effect occurs for the relative IBIs in (**c**) when compared to (**d**).

Next, I introduced a small cost to running resource deficits (*c* = 0.01) with a deficit limit of *λ* = 0.9. As illustrated in Fig. 3, unlike the case of *c* = 0, the relative fitness of offer strategies remained constant with increasing IBIs of *τ*_0_ = 270 and *τ*_0_ = 730. These results demonstrate that more equitable offer strategies can achieve higher relative fitness than less equitable strategies and that they do so by reducing resource variability and thereby reducing the costs of resource deficits.

### The evolution of equitable offer strategies

Because the relative fitness of equitable offer strategies played against themselves are greater than the relative fitness of less equitable offer strategies played against themselves, it should be possible for equitable agents to invade populations of non-generous agents if there is a mechanism that allows equitable agents to non-randomly assort. Population structure is a well-known mechanism that allows cooperators to assort^12^. Thus, to investigate whether equitable offer strategies could invade homogenous populations of non-generous agents, I ran simulations for populations structured by groups and, for controlled comparison, unstructured populations (see “Methods”, Agent-Based Model). All populations had a maximum size of *N* = 10,000 agents and groups randomly divided into two groups when they reached a size of 20 (see “Methods”, Agent-Based Model). There were 11 possible offer strategies ranging from 0.0 to 1.0 in increments of 0.1. An offspring inherited its parent strategy and mutations to other strategies occurred at rate *r* = 0.01.

**Figure 4:**
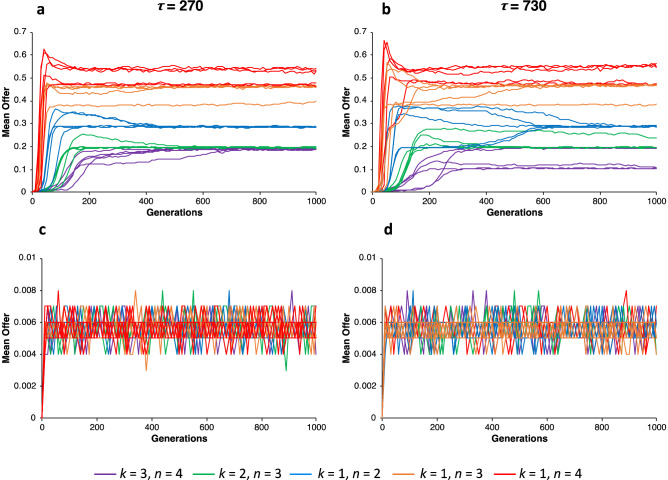
Invasion analysis of equitable offer strategies in group-structured and unstructured populations for*γ* = 0.9 and *c* = 0.01. Evolved mean offers in structured populations for *τ*_0_ = 270 (**a**) and *τ*_0_ = 730 (**b**) and for corresponding unstructured populations (**c**) and (**d**) respectively.

For the case of no cost (*c* = 0) for running a resource deficit, equitable offer strategies could invade non-generous populations for very short IBIs (*τ*_0_ = 10), but for longer IBIs, this effect rapidly diminished as expected (see Supplementary Fig. S4, for *τ*_0_ = 10, 100, 270). However, when there was a small cost to running resource deficits (*c* = 0.01) and the deficit-level cutoff was *λ* = 0.9, then for both *τ*_0_ = 270 and *τ*_0_ = 730, equitable offer strategies invaded populations of non-generous agents at similar levels (Fig. 4a,b) but they could not invade when populations were unstructured (Fig. 4c,d). A notable difference between *τ*_0_ = 270 and *τ*_0_ = 730 occurred for DG_3,4_. For *τ*_0_ = 270 mean offers evolved to the same level for both DG_2,3_ and DG_3,4_, but for *τ*_0_ = 730, three out of five of the simulated populations playing DG_3,4_ evolved to a lower than DG_2,3_. Nevertheless, in general, DGs with more recipients (DG_1,3_, DG_1,4_) evolved higher mean offers than DGs with only one recipient (DG_1,2_, DG_2,3_, DG_3,4_) and DGs with more dictators (DG_2,3_, DG_3,4_) evolved lower mean offers than DGs with only one dictator (DG_1,2_, DG_1,3_, DG_1,4_). These results align with the pattern of empirical data on congestible altruism (i.e., DGs with multiple recipients) and the bystander effect (i.e., DGs with multiple dictators).

Evolved mean offer should decrease when either the deficit-level cutoff, *λ,* is decreased or the time cost, *c,* to running a deficit is lowered. When the deficit-level cutoff, *λ*, was decreased, evolved mean offers decrease slightly (see Supplementary Fig. S5). For example, for *λ* = 0.85 and *λ* = 0.8 with *c* = 0.01, evolved mean offers across games slightly decreased (Supplementary Fig. S5). The largest relative drop for evolved mean offers was for DG_2,3_, which dropped from 19.5% on average for *λ* = 0.9 to 12% for *λ* = 0.8. Lowering the time cost (*c* = 0.005) also slightly lowered evolved mean offers (Supplementary Fig. S5). Thus, for both parameters, evolved mean offers gradually decreased (Supplementary Fig. S5) as the cost of running a resource deficit decreased to no cost at all (cf. Supplementary Figs. S4 and S5).

### Group size and offspring dispersion

**Figure 5:**
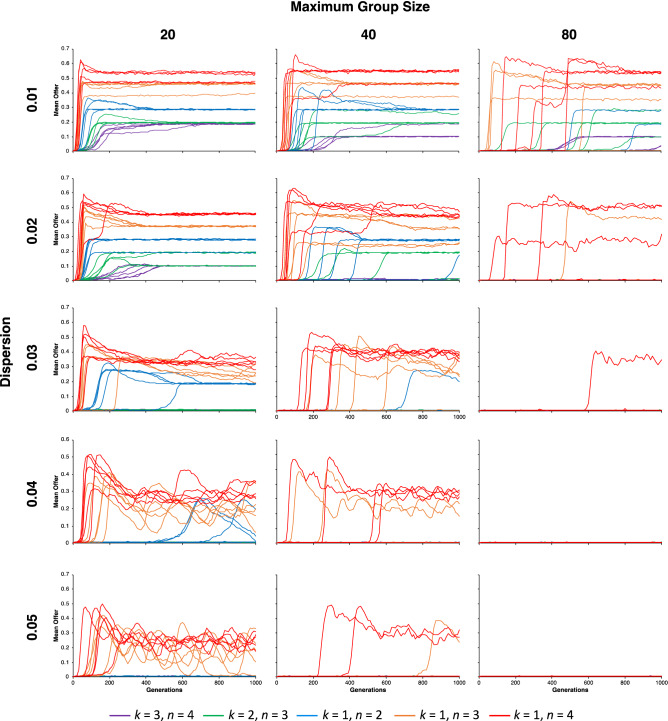
Invasion analysis of equitable offer strategies in group-structured and unstructured populations for *τ*_0_ = 270, *γ* = 0.9, and *c* = 0.01, where group size and offspring dispersal rates were systematically varied. As group size and offspring dispersal rates increased, the evolution of equitable offers decreased. The selection for equitable offers decreases most rapidly for multi-dictator games and least rapidly for multi-recipient games.

These effects clearly depend on ability of more equitable agents to non-randomly assort with each other, which can occur in viscous populations such as modeled here^25,26^. However, as group size and offspring dispersion increases, the ability of equitable offer strategies to invade should decrease. To assess how group size and offspring dispersion impacts the evolution of equitable offers, I systematically varied maximum group size (i.e., 20, 40, and 80) and offspring dispersion rates (i.e., 0.01, 0.02, 0.03, 0.04, and 0.05) for *τ*_0_ = 270. As illustrated in Fig. 5, equitable offer strategies were increasingly less likely to evolve as maximum group size increased or offspring dispersal increased. For a maximum group size of 80 and offspring dispersal rates greater than 0.03, even for multi-recipient games, equitable offer strategies could not invade.

Although equitable offer strategies could not invade when group size was too large or as offspring dispersal increased (Fig. 5), equitable offer strategies do have fitness advantages when they can assort with other equitable strategies. This opens up the possibility that other approaches could also facilitate the evolution of equitable offer strategies by increasing the likelihood of the assortment of equitable strategies. Such approaches could include strong reciprocity^27^, costly signaling^28^, and walking away from less equitable agents^29^.

## Discussion

Equitable offer strategies evolved in patterns consistent with those observed in *n*-person DG experiments. As the number of recipients increased relative to dictators, the size of total offers to all recipients increased and as the number of dictators relative to recipients increased, the size of offers decreased. This pattern of mean offers aligns with the pattern of results for congestible altruism (i.e., more recipients than dictators) and the bystander effect (i.e., more dictators than recipients) discussed above. Equitable offer strategies evolved because equitable distributions of resources reduced resource deficits and thereby achieved higher relative fitness. The evolution of equitable offer strategies also depended on non-random assortment of strategies (cf. Fig. 4a,b with c, d).

The psychological mechanisms that could have evolved to facilitate equitable behavior is a remaining challenge. One plausible candidate is self-centered inequality aversion^30–32^. There is evidence that people make equitable offers in DGs due to an aversion to inequality (or perhaps a preference for equality), but the model presented here also implies that an aversion to inequality is sensitive to social context. For example, an offer strategy of *p* = 0.2, should be much less averse to a dictator in a game with other dictators than it would be in a game with only multiple recipients (see Figs. 2 and 4 for change in relative variation as a function the number of recipients and dictators and evolved mean offers respectively). The results of this model imply that what is equitable changes with the number of participants and their roles, which aligns with mean offers reported in DG experiments on congestible altruism and the bystander effect.

These results also provide a theoretical explanation for why people are not entirely equitable and self-centered. Selection at the level of the group favored the evolution of offer strategies that maximized equitability among group members while selection at the individual level favored self-centered and less equitable offers. The mean offers that evolved were equitable but not the most equitable (cf. Figs. 2a,b and 4a,b). This suggests that psychological mechanisms could have evolved that are a mixture of concern for equitability among others and a self-centered concern for resource maximization. Although not investigated thoroughly here, the balance between concern for equitability and concern for self is likely modulated by environmental harshness, group size, and dispersion among groups. These considerations suggest that our theoretical understanding of the balance between concern for equitability and concern for self may need to include such ecological and population factors.

In hunter-gatherer societies, food sharing at the inter-family level can be viewed as *n*-person DGs. For example, food sharing among Ache foragers primarily occurs when one or a few families among a group are fortunate in acquiring an abundance of resources while hunting and gathering on a given day and they share them with other families who were not so fortunate^33^. Among the contemporary hunter-gathers Palanan Agta and Mbendjele BaYaka, food sharing occurs among clusters of families every 3 or 4 days on average^34^. This suggests that early humans could have acquired context-sensitive aversion to inequality that would allow them to equitably distribute resources for different combinations of those in need and those who can contribute as in *n*-person DGs.

If *n*-person DGs can be used to model sharing in hunter-gatherer groups, this raises interpretational issues. What are the units (e.g., individual, families) of participation in a DG? A tacit assumption of this model is that the unit is all the individual members of a group. However, hunter-gatherer groups are complex systems that consist of multiple levels of organization that are hierarchical in structure and may have evolved to maximize the efficient distribution of resources to reduce deficits^35^. Thus, units that play DGs may vary depending on the level of organization. Another issue is the time interval between sharing events. This model tacitly assumed that DGs were played on a daily basis. As mentioned above, DG-like sharing events could occur on longer time scales such as every several days or more. If this model is to provide insight into human equitability, then much more theoretical work is required to understand how equitability could have evolved in complex human social systems with multiple levels of organization and time scales.

IBIs are one kind of time constraint on the evolution of sharing, but there are others. For example, common vampire bats can only survive 60 h without a blood meal^36,37^. This introduces a constraint on survival analogous to IBIs, which foraging variability can greatly affect. Vampire bats are very successful at foraging for blood meals, but if unsuccessful for two nights in a row, a starving female can only survive if she receives a blood meal from other females in her social group. Interestingly, common vampire bat populations are subdivided into small stable social groups (consisting of 8–12 adult females) with low migration between social groups^37^, which when combined with the 60-h survival constraint without a blood meal create conditions similar to the models developed here that favored the evolution of equitable offers. Vampire bats also illustrate the importance of modeling resources for survival and if resource-based survival is incorporated into fecundity-based models—such as the one presented here—this would enhance selection for equitable offer strategies.

Binmore has argued that blood meal sharing in vampire bats is a kind of implicit insurance contract that has evolved to avoid the deadly resource deficits that rapidly lead to starvation^38^. An implicit insurance contract interpretation of sharing could, with the help of cultural evolution, underlie fairness and moral principles in human societies^38^. Here equitable offers could be interpreted as context-dependent implicit insurance contracts that reduce resource deficits and thereby increase the rate of reproduction of equitable agents. As discussed above, the multilevel selection pressures favoring and opposing equitable behaviors could explain an evolved context-dependent aversion to inequality, which could then provide psychological foundations for fairness and moral principles in humans societies.

A coevolutionary explanation for inequality aversion in humans and other animals has been proposed by Brosnan^39,40^. On this hypothesis, inequality aversion is advantageous because it promotes sharing of payoffs from cooperative interactions and thus coevolves with cooperation as a mechanism for facilitating cooperation. The results presented here are consistent with this hypothesis but more importantly demonstrate that inequality aversion could evolve directly from the fitness benefits of equitable distributions of resources. As with all models, this model is highly simplified representation of a very simple game. Nevertheless, fruitful models point the way to new directions for theoretical development. This suggests that accounting for both the synergistic payoffs of cooperation and their equitable distribution may shed new light on the evolution of cooperation.

## Methods

### Resource variation

Assuming homogenous DG_*k*,*n*_ populations of agents all playing the same offer strategy *p*, the variance in resources for an agent on each time step is calculated by summing the squared deviations from the mean, *kR*/*n*, for two components: the probability, *k*/*n*, of being a dictator plus the probability, (*n* − *k*)/*n*, of being a recipient. As a dictator, an agent keeps *R* − *pR* and as a recipient, receives *kpR*/(*n* − *k*). The variance for an offer strategy for a given time step is the proportion of variance contributed when playing the dictator role plus the variance contribution when playing the recipient role as expressed in Eq. (9)

9 $${\sigma }_{p}^{2}=\frac{k}{n}{\left(\frac{kR}{n}-(R-pR)\right)}^{2}+\frac{\left(n-k\right)}{n}{\left(\frac{kR}{n}-\frac{kpR}{n-k}\right)}^{2}$$

which simplifies to Eq. (10).

10 $${\sigma }_{p}^{2}=\frac{k{R}^{2}{\left(k+np-n\right)}^{2}}{{n}^{2}(n-k)}.$$

Because whether an agent is a dictator or recipient is independent between time steps, the variance for accumulated resources over *τ*_0_ time steps is the sum of the variance for *τ*_0_ time steps, which is given by Eq. (11).

11 $${\sigma }_{p,{}_{0}}^{2}={}_{0}\frac{k{R}^{2}{\left(k+np-n\right)}^{2}}{{n}^{2}(n-k)}.$$

Finally, the coefficient of variation in Eq. (4) above for accumulated resources over *τ*_0_ time steps is the square root of Eq. (11) divided by the expected accumulation of resources over *τ*_0_ time steps, *τ*_0_*kR*/*n*.

### Simple agent model

The aim of this model was to compute the relative fitness for offer strategies, *p*, played against themselves. Pseudocode for this model is provided in the Supplementary Table S1. For all DG_*k*,*n*_ simulations, the expected accumulation of resources was held constant for each time step by setting the expected payoff in resources to a constant $\mu$ = *kR*/*n* and the resource endowment for each DG_*k*,*n*_ was

12 $$R =\frac{n\mu }{k}.$$

Thus, the expected payoff per agent was held constant across all DG_*k*,*n*_.

Gaussian variation was also introduced into the game resource endowment for a dictator as specified by Eq. (13)

13 $${R}_{i,t}=R+\mathrm{GAUSS}\left(\right)\times {\sigma }_{R},$$

where ${R}_{i,t}$ is the resource endowment for the *i*th dictator on time step *t* with ${\sigma }_{R}=0.1R$. That is, the quantity of resources provided a dictator was randomly drawn from a Gaussian distribution with mean *R* and standard deviation ${\sigma }_{R}$. This adds realism by allowing endowed resources to vary around *R* and it avoids artifacts that can occur when *R* is a multiple of the threshold for accumulated resources.

Agents had fixed lifespans. Lifespan expiration was the only way agents were removed from the simulation. Fitness was based solely on fecundity and not resource-based survival. Agents had an average lifespan of *ω*, which varied for each agent *i* according to Eq. (14)

14 $${\omega}_{i}=\mathrm{trunc}\left(\omega+\mathrm{GAUSS}\left(\right)\times {\sigma }_\omega\right),$$

where *ω* = 3*τ*_0_ so that on average an agent could optimally reproduce three times during its lifetime if no variation is introduced into agent lifespans. However, for *σ*_*ω*_ = 0.25ω, the expected number of offspring per agent is approximately 2.5 offspring per agent. At birth, an agent’s age was set to zero and incremented by one on each time step. When an agent *i* reached the age *ω*_*i*_, it was removed from the simulation. For example, for *τ*_0_ = 270, agents on average lived 810-time steps and for *τ*_0_ = 730, agents on average lived 2,190-time steps.

For all simulations using this simple agent model, 100,000 agents were simulated for parameter conditions listed in Table 1. For each agent, the number of offspring born during its lifespan was recorded as well as the number of time steps between each IBI. The relative fitness of each offer strategy was calculated by dividing the mean number of offspring born for each offer strategy *p* by the grand mean (i.e., the mean number of offspring born for all eleven offer strategies) of the different offer strategies for each DG_*k*,*n*_. Relative IBIs for each offer strategy were calculated by dividing the mean length of IBIs for an offer strategy *p* by the grand mean (i.e., the mean length of IBIs for all eleven offer strategies) of the different offer strategies for each DG_*k*,*n*_.

**Table 1:** Parameters and their interpretation used in the simple agent model and the agent-based model.

Parameters	Values and parameter sweep conditions
DG_*k*,*n*_	Five DG_*k*,*n*_ for all simulations: DG_3,4_, DG_2,3_, DG_1,2_, DG_1,3_, and DG_1,4_
*p*	Offer strategy and for all simulations, 11 offer strategies were simulated in the range 0.0, …, 1.0 in increments of 0.1
*R*	Base resource endowment for dictators was set to *R* = *μn*/*k*, where *μ* = 5. For each dictator, random variation was introduced by Eq. (13)
*σ*_*R*_	Standard deviation in resource endowment, and *σ*_*R*_ = 0.1*R* for all simulations
*τ*_0_	The optimal IBI was set to 270 and 730, where 270 is approximately the length of human gestation and 730 is two years
*I*_*o*_	Minimum resources required to reproduce was set to *I*_*o*_ = *μτ*_0_*,* where *μ* = 5
*ω*	The mean lifespan of an agent was set to *ω* = 3*τ*_0_, which allowed agents to reproduce approximately three times during a lifetime. Random variation was added to each agent’s age as specified in Eq. (14)
*σ*_*ω*_	The standard deviation in lifespan of an agent was set to *σ*_*ω*_ = 0.25ω for all simulations
*r*	Mutation rate was set to 0.01 for all simulations
*d*	Dispersal rate of offspring was set to 0.01 and varied
*γ*	Resource deficit-level cutoff for the expected accumulation of resources with *γ* = 0.9 and varied
*c*	The time cost, where *c* = 0.01 and varied

### Agent-based model

The full ODD description of the model is in the Supplementary Information^41^. The simple agent model above was expanded to a population model. For all simulations, a population consisted of *N* = 10,000 agents. The length of a simulation was the mean lifespan, *ω*, times 1000, which corresponds to an average of 1000 generations for all simulations. For structured populations, groups were formed by subdividing the initial population of 10,000 agents into 1000 groups of 10 non-generous (i.e., *p* = 0) agents each and randomly placing the groups in a 100 × 100 toroidal grid space. Groups could randomly fission into two approximately equally sized groups once they reached 20 members. One group remained at the parent’s group location and the other was placed in a randomly selected cell elsewhere in space. If a group contained no agents, it was removed from the simulation. As a simulation progressed, the average number of groups increased to approximately 1317 with a mean group size of 7.6 for a maximum group size of 20. For a maximum group size of 40, there were on average 708 groups with a mean group size of 14.1 and for a maximum group size of 80, there were on average 374 groups with a mean group size of 26.7. In all cases, group sizes varied during a simulation between one and the maximum group size.

Resource endowment and lifespan were calculated using Eqs. (13, 14). In group-structured populations, all agents participating in DGs were selected from the same group (see illustrative Fig. 1) and for unstructured populations, agents were selected from the entire population. For each time step, dictators and recipients were sorted into two lists and their order randomized. Each dictator played the required number of recipients by incrementing through the recipient list. If the end of the recipient list was reached with more recipients required, the order of the recipients in the list was again randomized and the remaining dictators selected recipients from the top of the recipient list again proceeding incrementally through it. This procedure was repeated until all dictators had played the required number of recipients for a DG_*k*,*n*_.

This procedure did not guarantee that all agents in a group participated in a DG_*k*,*n*_ on a given time step. Four types of cases could occur by chance all of which increased resource variability but preserved the expected return of *kR*/*n* per time step. First, there could be no dictators in a group (this is most likely to occur in small groups) in which case, all agents in the group received no resources. Second, there could be too few dictators in a group for all recipients to participate in a game in which case, some recipients received no resources. Third, there could be no recipients in a group (this is most likely to occur in small groups) for dictators to play in which case, dictators kept all of their endowed resources for that time step. Fourth, there could be too few recipients in a group in which case, some recipients participated in more than one DG on a given round of play. Each of these cases increased resource variability, which introduced a small bias against the evolution of equitable offers. These cases were relatively negligible for unstructured large populations.

The IBI *τ* was updated for each agent according to Eq. (6) and if condition (7) was satisfied (see illustrative Fig. 1), an agent successfully produced an offspring if the population was under its maximum size *N* = 10,000. A parent agent’s reproductive resources were then reduced according Eq. (9). When an agent was born, it randomly mutated at rate *r* = 0.01 to another offer strategy from the range 0.0 … 1.0 in increments of 0.1 and entered the population if there were less than *N* agents otherwise it “died”. At birth, offspring dispersed at rate *d* = 0.01 to another randomly selected group, if available, within 100 × 100 grid of cells composing the agent’s 2-D space. Agents were removed from the population when their lifespan expired and this was the only way agents were removed. For the parameters used in the invasion analyses see Table 1.

## Supplementary Information

Supplementary Information.

## Data Availability

The computational models used are available at https://schanklab.repositoryhosting.com/trac/schanklab_ndg/browser.
