# Supplementary material for: A model of the evolution of equitable offers in n-person dictator games with interbirth intervals
Source: Sci Rep. 2021 Jul 30;11:15544. doi: 10.1038/s41598-021-94811-3 (PMC8324864; doi:10.1038/s41598-021-94811-3)
Supplement: Supplementary file 1 — Supplementary Information. [file 41598_2021_94811_MOESM1_ESM.docx]

Supplementary Material

**A model of the evolution of equitable offers in n-person dictator games with interbirth intervals**

Jeffrey C. Schank

Department of Psychology, University of California, Davis,

Davis, CA, United States

email: jcschank@ucdavis.edu

| **Table S1**. Pseudo code for the simple agent model. |
| --- |
| INITIALIZE k = 1, 2, 3 (varies with simulations; the number of dictators)  INITIALIZE n = 2, 3, 4 (varies with simulations; the total number of participants in a game)  INITIALIZE ibi = 270, 730 (minimum ibi, varies with simulations)  INITIALIZE baseResource = 5 (arbitrary constant)  INITIALIZE R = baseResource × n/k (resource endowment for a DGk,n)  INITIALIZE resourceEndowmentSuccessRate = k/n (frequency of dictators)  INITIALIZE Io = baseResource × ibi (quantity of resources required to reproduce)  INITIALIZE maxReproductiveBirths = 3 (The maximum number of births)  INITIALIZE averageAge = maxReproductiveBirths × ibi (the average age of agents)  INITIALIZE averageAgeSD = 0.1 × averageAge (standard deviation in maximum age)  INITIALIZE p = (offer value in the range 0.0, ... 1.0 in increments of 0.1 and varies with simulations)  INITIALIZE deficitLevel = 0.9 (the level below which a time cost is imposed)  INITIALIZE ibiCost = 0.01 × ibi (the cost of running a deficit)  INITIALIZE expectedResources = R × k/n  FOR i = 1 to 100000  maxAge (the maximum age of agent) = averageAge + GAUSS × averageAgeSD  resources = 0 (starting resource of agent)  ibiStep = 1 (starting IBI incremental variable for agent)  ibiVar = ibi  FOR j = 1 to maxAge  Endowment = R + GAUSS × 0.1 × R (endowment is R plus Gaussian variation)  IF (RANDOM ≤ resourceEndowmentSuccessRate)  resources = resources + Endowment – [p × Endowment] (amount kept as  dictator)  OTHERWISE  resources = resources + p × Endowment × k/[n - k] (amount received as recipient)  IF (resources < (expectedResources × ibiStep × deficitLevel))  ibiVar = ibiVar +ibiCost (impose cost for running a deficit)  IF ibiStep ≥ ibi AND resource ≥ Io  REPRODUCE: add 1 to reproductive count of offspring produced  IBI: record actual ibiStep, which may be > minimum IBI  ibiStep = 0 (since if is incremented by 1 immediately, it is set to 0 here)  ibiVar = ibi (reset to ibi)  resources = resource - Io (cost of reproducing)  END IF ibi  ibiStep = ibiStep + 1 (increment the ibiStep variable)  END FOR j  Record number of offspring produced by the ith agent.  END FOR i |

**Overview, Design concepts, and Details (ODD): Agent-based model**

## **Purpose**

The model is used to investigate whether equitable offers can evolve when agents have interbirth intervals (IBIs) in populations structured by groups. While population structure is a well-known mechanism for the evolution of cooperation,^1^ this model aims to show that IBIs introduce a constraint on the rate of reproduction, which allows resource variability to modulate fitness in ways that favor the evolution of equitable offers by reducing resource deficits.

## **State Variables and Scales**

The model contains two types of entities: agents and groups. Agents are characterized by the offers they make when playing *n*-person DGs (DG*_k_*_,_*_n_*), reproductive state (which consists of accumulated resources and IBIs), age and lifespan, dispersal rate, and mutation rate of offer strategies during reproduction. Groups consist of agents and groups are located at *i*,*j*- locations within a 100 × 100 grid of cells with toroidal boundaries. Within groups, agents play a DG*_k_*_,_*_n_* with other randomly selected members in their group. A DG*_k_*_,_*_n_* is characterized by dictators *k* and the total number of participants *n* with *n* – *k* recipients. Groups fission when they reach the maximum size of 20 (or 40 and 80 in group-size simulations) and do so by randomly dividing into two approximately groups (i.e., each agent in a group, it either stays in the parent group or is placed in the offspring group). Group extinction occurs when a group has no members. Offspring groups are placed in a randomly selected empty cell in the 100 × 100 grid of cells.

## **Process overview and scheduling**

Initialization

At the beginning of a simulation, agents were initialized as described in **Table S2**. In all simulation, 1,000 groups were created, each containing 10 agents, and randomly placed in a 100 × 100 discrete space. For all invasion simulations, the initial offer strategy was *p* = 0. **Figure S1** illustrates an example of an initial random configuration of groups with subsequent snapshots of simulation states. As simulations progressed, the number of groups on a given step increased from 1000 to an average of about 1317 for an average groups size of 7.6 with a range of 1 to 20.

Scheduling and Agent Behavior

All agents were placed in MASON's simulation schedule and the behavior of agents was executed by class. First, on each time step, all the agents were stepped in random order as described in **Table S3** and illustrated in **Figure S2**. Next all of the groups of agents were stepped in random order as described in **Table S4** and also illustrated in **Figure S2**. Finally, the last class of agent stepped was the experimenter agent, which collected and calculated the data as described in the **Observation** section below.

**Figure S1**. Snapshots from a simulation using the parameter values for *τ*_0_= 270 (see **Table 1** of the manuscript). The top figure is a chart from MASON illustrating the change in mean offer for a single run with one dictator and one recipient. The bottom set of panels are snapshots of groups distributed in space. Starting from the top left panel at initialization, groups were randomly dispersed in space. From the middle top panel and wrapping around to the bottom row of panels, are snapshots from steps 0, 202,500 (250 generations), 405,000 (500 generations), 607,500 (750 generations), and 810,000 (1,000 generations) with corresponding arrows drawn in the top chart to indicate the snapshots correspondence to evolved mean offers. Groups are color coded by the average offer strategies of the agents in them. Red was the non-generous offer, *p* = 0. Offers became increasingly purple as offers became increasingly equitable and offer became increasingly blue as generosity increased past 0.5.

**Figure S2**. Illustration of the behavior of agents at individual and group levels on each time step *j*. Pseudo code describing the behaviors in detail is provided in the referenced tables. At the individual level, an agent's age is checked first. If its lifespan has expired, it “dies” else it can reproduce (see **Tables** **S5** and **S6**), and then its age is incremented by 1. For groups, if there are no members, the group dissolves (**Table** **S4**). If there are members, the DG*_k_*_,_*_n_* is played as described in **Table S8** below.

| **Table S2**. Initialize of simulations. |
| --- |
| INITIALIZE N = 10000 (initial number of agents in a group)  INITIALIZE maxN = 10000 (maximum number of agents in a simulation)  INITIALIZE groups = 1000 (initial number of groups for populations with structure)  INITIALIZE maxGroupSize = 20 (number varies with simulations)  INITIALIZE minGroupSize = 0 (minimum group size without group dispersal)  INITIALIZE groupRadius = 100 (specifies a 100 x100 grid, offspring groups are placed in empty cells within this grid)  INITIALIZE mutationRate = 0.01 (mutation rate for offers)  INITIALIZE ibi = 270, 730 (varies with simulations)  NITIALIZE baseEndowment = 5 (resource endowment mean used calculate resource endowment based on the number of dictators)  NITIALIZE Io= ibi x baseEndowment (resources required to reproduce)  INITIALIZE averageAge = Io x 3 (on average, 3 reproductive cycles)  INITIALIZE sdAge = 0.25 (as a percent of the averageAge)  INITIALIZE n_k = 1 (the number of recipients, varies with simulations)  INITIALIZE k = 1 (the number of dictators, varies with simulations)  INITIALIZE n = k + n_k (total number of participants in a game)  INITIALIZE R = baseEndowment × n/k (The base resource endowment for dictators)  INITIALIZE RSD = 0.1 (standard deviation in resource endowment)  INITIALIZE resourceEndowmentSuccessRate = k/(k + n_k) (success rate at receiving an endowment)  INITIALIZE dispersalRate = 0.01 (dispersal rate, varies with simulations)  INITIALIZE dispersalRadius = 1, (agents can disperse to any group in 3 x 3 grid)  INITIALIZE deficitLevel = 0.9 (the level below which a time cost is imposed)  INITIALIZE ibiCost = 0.01 x ibi (the cost of running a deficit)  INITIALIZE expectedResources = R x k/n  FUNCTION INITIALIZE_SIMULATION  FOR i = 1 to number of groups  CREATE: Group g  FOR j = 1 to the number of agents in a group,  CREATE: Agent a  SET_LIFESPAN: calculate a lifespan for agent a  INITIALIZE_AGE: (lifespan): calculate an initial age for a  INITIALIZE_RESOURCES: initialize starting resources to 0  INITIALIZE: ibiStep = 1  INITIALIZE: ibi = 270, 730 (minimum ibi, varies with simulations)  INITIALIZE: *p* = 0 (initialize offer strategy to 0)  END FOR LOOP  END INITIALIZE_SIMULATION    FUNCTION SET_LIFESPAN  *lifespan* = (Integer)(averageAge + GAUSS() × sdAge × averageAge )  RETURN *lifespan*  END SET_LIFESPAN  FUNCTION INITIALIZE_AGE (argument lifespan)  RETURN *age* is set to a random integer in the range 0 to *lifespan* – 1  END INITIALIZE_AGE |

| **Table S3**. Agent step method. |
| --- |
| FUNCTION STEP  CHECK AGE: Determine if agent dies  INCREMENT: ibiStep + 1 (time since last birth)  CHECK REPRODUCTION: see **Table S5**  CHECK GROUP FISSION: IF agent reproduces, see **Table S7**  INCREMENT: age + 1  END STEP |

| **Table S4**. Group step method. |
| --- |
| FUNCTION STEP  CHECK IF GROUP IS EMPTY: IF the group is empty, group dissolves  PLAY a DG: see **Table S8**  END STEP |

| **Table S5**. Reproduction. |
| --- |
| FUNCTION REPRODUCE  IF (resources ≥ *Io* AND ibiStep ≥ *ibi*),  IF the total number of agents is less than the maximum number of agents,  CREATE: offspring clone of parent  MUTATION: random mutations are introduced at rate *r* for the sharing  parameter from the interval [0.0, 0.1, …, 1.0]  AGE: age at birth is set to 0  Offspring IBI: *ibiStep* = 1  REPRODUCTIVE_RESOURCES: resources set to 0 for offspring  IF (RANDOM: probability ≤ disperse probability), THEN  DISPERSE: offspring disperses to another group, see **Table S6**  ELSE  ADD: offspring to parent group  RESET_RESOUCES: parental resources to resources – *Io*  RESET: *ibiStep*= 1  ELSE  RESET_RESOUCES: parental resources to resources – *Io* RESET: *ibiStep*= 1  END REPRODUCE |

| **Table S6**. Dispersal. |
| --- |
| FUNCTION DISPERSE  LOCATE: find a randomly selected group within in space  IF (a group is found that is not its current group)  ADD: agent to the new group  ELSE  do nothing, agent remains in current group  END DISPERSE |

| **Table S7**. Group fission. |
| --- |
| FUNCTION FISSION  IF (number of members ≥ *maxGroupSize* AND there is an empty location within the fission radius of the parent group), THEN  CREATE: an offspring group, it is placed in the offspring group with probability of 0.5, otherwise it remains in the parent group  PLACE: the offspring group is placed in a randomly selected empty  location within a 100 x 100 grid cells  END FISSION |

| **Table S8**. Implementation of DG*_k_*_,_ *_n_*. |
| --- |
| FUNCTION GROUP_PLAY_DG  FOR each group member i, from 1 to the number of agents in the group  IF (random probability ≤ *p*), THEN  i’s current resources = *R* + *R* x GAUSS x *RSD* (agent receives a resource endowment)  agent i is added to the list of *dictators*  ELSE  i’s current resources = 0 (agent does not receive a resource)  agent i is added to the list of *recipients*  ADD: i = i + 1  END FOR LOOP  IF (*dictators* is empty OR *recipients* is empty), THEN  END GROUP_PLAY_DG (since there are no games to play).  RANDOMIZE: the order of *recipients*  RANDOMIZE: the order of *dictators*  INITIALIZE: j = 1  FOR each agent i in *dictators*, from 1 to the number of agents in *dictators*,  *x* = [*p* x (current resources)]/(*n* – *k*)  OFFER: i offers an amount *x* of its current resources to *n* – *k* recipients  ADD: i adds to its total resources (current resources – *x*) AND the next *n* – *k*  recipients add *x* resources to their total resources  IF (j > the number of *recipients*), THEN (the end of the list of recipients is reached)  RANDOMIZE: *recipients* (when all the *recipients* have  played the DG, the order of recipients is randomized and the above  procedure is repeated until all of the dictators have played)  SET: j = 1  ADD: j = j + *n* – *k* (the count of the number of recipients is incremented *n* – *k* after  each play with a dictator i  END FOR LOOP  END GROUP_PLAY_DG |

## **Design concepts**

Basic Principles

A working hypothesis of this model is that IBIs are crucial for the evolution of equitable offers in DGs. Following optimal resource allocation theory,^2^ the optimal number of offspring that an agent can produce is the total resources available for reproduction divided by the resources required to produce an offspring as described in the manuscript.

Equitable offers can only evolve if they can assort non-randomly and populations subdivided into groups is one mechanism for non-random assortment. Agents obtain resources by playing a DG*_k_*_,_*_n_* and resources are accumulated over time to reproduce offspring. The rate of reproduction is constrained by IBI *τ*_0_. This approach allows groups of agents who share resources by making equitable offers to more efficiently distribute resources among agents within the group and thereby increase the reproductive rates by reducing resource deficits and thereby minimizing IBIs (i.e., minimizing actual number of time steps between births which may be longer than *τ*_0_).

Emergence

Two aspects of this model were emergent and of particular interest. First, mean offers were emergent from these evolutionary dynamics. Second, the relative fitness of an agent emerged from group-population structure as well as the actual IBIs.

Stochasticity

Stochasticity was introduced at all levels of simulations. The order in which agents and groups are stepped in simulations is randomized using the MASON schedule. The assignment of agents as dictators and recipients was random. The resource endowment each dictator receives was randomized. Dispersal at birth, initial location of groups, and when groups fission was random. Lifespans were randomized. Mutations were random.

Observation

For all sets of parameter values, five simulations were run and the mean offers from each simulation was computed.

Initialization

At the start of a simulation, 10,000 agents were created and assigned the non-generous offer strategy *p* = 0 (see **Table S2**). For unstructured populations, all agents were assigned to one group and for structured populations, 10 agents were assigned to each of 1000 groups and randomly placed in unique locations in the 100 × 100 grid space with toroidal boundaries (see **Fig. S1**). Lifespans and resources are described in **Table S2**.

## **Submodels**

Submodel Resource Endowment

The resource endowment for a game (DG*_k_*_,_*_n_*) is *R* and is defined by equation (12) in the manuscript. Random Gaussian variation was introduced into each dictator's endowment (see equation 13 of the manuscript). The pseudocode for implementation endowed resources is provided in **Table S8**.

Submodel DG*_k_*_,_ *_n_*

On each time step, approximately *k*/*n* portion of agents were randomly selected to receive a resource endowment *R* as specified in (**Table S8**). These were the dictators for a given time step. The remaining (*n* – *k*)/*n* portion of agents were recipients. The recipients were randomly ordered and each dictator played exactly (*n* – *k*) recipients, where each recipient was played in order until the end of the list of recipients was reached. If there were still more dictators left to play, the list of recipients was randomized again and this process was continued until each dictator had played exactly (*n* – *k*) recipients. The pseudocode implementation for this submodel is provided in **Table S8**.

Four types of situations can occur either there are no dictators in a group, too few dictators in a group, no recipients in a group, or too few recipients. These cases are discussed in the manuscript. These cases increase resource variability and should tend to favor non-equitable offer strategies.

### Submodel Lifespan

### Each agent had a fixed lifespan ${}_{i}$ characterized by a mean and standard deviation $\sigma$ (equation 14 in the man text). The average lifespan of an agent was set to allow approximately three births during an agent's lifespan. Thus, mean lifespan *ω* was always = 3*τ*_0_, where *τ*_0_ was the minimum IBI. The pseudocode implementation for this submodel is provided in **Table S2**.

Submodel Reproduction

A birth required the accumulation of at least *I_o_* = *τ*_0_*kR*/*n* units of resources and a minimum of *τ*_0_ time steps between births (or their own birth). The parameter *τ*_0_ is the minimum IBI. Agents could produce at most one offspring agent per birth. The total number of agents in a population was capped at agents *N* = 10,000. If the number of agents in the population was less than 10,000, then a birth was successful and the offspring agent entered the population, otherwise the birth was not successful. When a *birth* (see equation 8 in the manuscript) occurred, $I_{o}$ resources were subtracted from the agents current accumulated resources (see equation 9 in the manuscript).

Submodel Inheritance and Mutation

Agents have one heritable phenotype and that is their offer strategy, which is one of 11 possible real values in the range *p* = 0.0, …, 1.0 in increments of 0.1. Reproduction was haploid and so offspring inherited the offer strategies of their parent unless a mutation occurred with probability *r* = 0.01. If a mutation occurred, an offspring’s offer strategy was drawn from the range of 11 possible values excluding the parent strategy.

Submodel Dispersion

At birth, offspring agents disperse from their parental group to another group (if one exists) in the 100 × 100 grid of cells with probability *d*. As the dispersal rate *d* increased, populations effectively become panmictic. The pseudocode implementation of this submodel of dispersion is provided in **Table S6**.

Submodel Group Fission

Populations were subdivided into groups with a maximum size of 20 (or 40 and 80 for group size simulations). When a group reached 20 (or 40 and 80 for group size simulations), it fissioned into two groups. The parent and offspring group were randomly divided into two groups, which on average were of equal size but varied binomially. The parent group remained at the current location and the offspring group was located (if a space is available) within the 100 × 100 grid centered on the location of the parent group. The pseudocode implementation of this submodel of group fission is provided in **Table S7**.

**References**

1. Apicella, C. L. & Silk, J. B. (2019) The evolution of cooperation. *Current Biology* **29**, R425-R450.
2. Smith, C. C & Fretwell, S. D. (1974) The optimal balance between size and number of offspring.

|  |
| --- |
| **Figure S3**. The effects of accumulated resource variation on the relative fitness (i.e., the relative fitness of *p* is calculated by dividing *p* by the mean fitness of all *p* for each DG*_k_*_,_*_n_*) and on the relative IBIs (i.e., the IBI of *p* divided by mean *p* for each DG*_k_*_,_*_n_*) of offer strategies for five DGs. While plots of relative fitness display similar forms for *τ*_0_ = 10 (**a**) and *τ*_0_ = 270 (**b**), the range of relative fitness has is much smaller for *τ*_0_ = 270 than for *τ*_0_ = 10. A similar effect occurs for the relative IBIs (**c**) when compared to (**d**). |

|  |
| --- |
| **Figure S4**. Invasion analysis of equitable offer strategies for no cost (*c* = 0) for running a resource deficit. As the length of IBIs increased from *τ*_0_ = 10 to *τ*_0_ = 270, evolved mean offers decreased sharply and the evolved mean offers for all five DG*_k_*_,_*_n_* converged (see Fig. S3 for the corresponding decrease in relative fitness). |

|  |
| --- |
| **Figure S5**. Invasion analysis of equitable offer strategies for group-structured populations with different deficit and cost levels. For all three sets of simulations, *τ*_0_ = 270. The top two graphs illustrate evolved mean offers over generations for deficit levels of *γ* = .85 and .8 respectively with *c* = 0.01. The bottom graph depicts evolved mean offers over generation for a cost *c* = 0.005 with a deficit level of *γ* = .9. |
